# Personalization of Neoadjuvant Immunotherapy in High-Risk Resectable Melanoma and Utility of ctDNA as a Biomarker of Immunotherapy Response

**DOI:** 10.1245/s10434-026-19814-8

**Published:** 2026-05-23

**Authors:** Aleigha Lawless, Derek N. Effiom, Alma Burbano, Tanya Sharova, Thomas J. Otten, Madak Basnet, Emily Spurr, Tarini Shankar, Maria Christina Crespo, Julianne Andrade Czapla, Gina Bangrazi, Kevin Emerick, Ryan J. Sullivan, Genevieve M. Boland, Meghan J. Mooradian, Sonia Cohen

**Affiliations:** 1 Department of Surgery, Mass General Brigham, Boston, MA USA; 2 Mass General Brigham Cancer Institute, Boston, MA USA; 3 Mass Eye and Ear, Boston, MA USA; 4 Harvard Medical School, Boston, MA USA

**Keywords:** Neoadjuvant Immunotherapy, Pathologic Response, Circulating tumour DNA, Survival Outcomes

## Abstract

**Background:**

Randomized trials demonstrated superior outcomes with neoadjuvant immune checkpoint inhibitors (ICI) compared with adjuvant ICI alone for patients with resectable macroscopic melanoma. However, the optimal perioperative strategy remains undetermined, and biomarkers are lacking. Real-world data reporting the feasibility of limited resection and the utility of circulating tumor DNA (ctDNA) in predicting melanoma recurrence could help inform optimal practice.

**Methods:**

Retrospective data regarding treatment, outcomes, and safety were collected for 76 patients who had melanoma treated with neoadjuvant ICI from 2020 to 2024. Signatera ctDNA results were available for 22 patients.

**Results:**

Of the 76 patients, 42 (55%) received ipilimumab plus nivolumab (Ipi/Nivo), 31 (41%) received anti-PD1 monotherapy, and 3 (4%) received nivolumab plus relatlimab. The patients included 64 (84%) who underwent planned surgical excision, 25 who underwent upfront total lymph node dissection (TLND), and 32 who underwent index node excision (INE), with reflex TLND performed for 7 patients. The overall major pathologic response (MPR) rate was 55% (49% with Ipi/Nivo and 65% with monotherapy). Six (21%) of 28 non-MPR patients did not receive adjuvant therapy. After a median follow-up period of 15.8 months, recurrence rates were comparable between the INE (9%) and TLND (12%) cohorts. At the time of surgery, ctDNA was undetectable in the majority of the MPR patients (9/11) and the minority of the non-MPR patients (4/11). Postoperatively, ctDNA was detectable in 1 of 20 patients.

**Conclusions:**

A personalized surgical approach to neoadjuvant ICI was feasible, with comparable recurrence rates between the patients who underwent INE and those who underwent TLND. For the patients without MPR, subsequent adjuvant therapy improved recurrence-free survival (*p *= 0.046). The ctDNA results correlated with the clinical outcomes, suggesting that ctDNA may complement pathologic response in guiding management.

**Supplementary Information:**

The online version contains supplementary material available at 10.1245/s10434-026-19814-8.

Melanoma is an aggressive malignancy, and although it represents a small percentage of skin cancers, it accounts for the most deaths.^1^ Until recently, surgery has been the primary treatment for macroscopic, resectable disease. However, patients remain at high risk of recurrence even after complete resection. Adjuvant immune checkpoint inhibition (ICI) and targeted therapy were approved for treatment of stage III melanoma in 2017 and 2018 after seminal trials demonstrated improved recurrence-free survival (RFS) and distant-metastasis free survival (DMFS) compared with surveillance alone.^2–5^ Despite these advances, up to 50% of patients do not benefit from adjuvant therapies, and we lack biomarkers to predict those patients who will benefit from treatment.^6^

Neoadjuvant administration of ICI proved superior to adjuvant ICI in recent practice-changing studies and currently is the standard of care for macroscopic, resectable melanoma.^7–9^ Randomized clinical trials have shown that neoadjuvant ICI improves event-free survival (EFS) compared with adjuvant ICI alone. For example, the phase 2 SWOG-1801 trial reported a 23% improvement in EFS with perioperative anti-PD1 therapy compared with the same planned therapy given exclusively in the adjuvant setting.^8^

In addition to improving survival rates, a neoadjuvant approach allows assessment of pathologic response, which has been shown to strongly correlate with clinical response.^7,8,10^ The phase 3 NADINA trial reported longer EFS with neoadjuvant flipped-dose ipilimumab (anti-CTLA4) plus nivolumab (anti-PD1) than with adjuvant nivolumab alone. This trial used pathologic response to de-escalate adjuvant therapy for patients with a major pathologic response (MPR), defined as ≤ 10% viable tumor, after neoadjuvant ICI. Despite omission of adjuvant therapy, patients with an MPR had a 12-month RFS of 95.1%, compared with 76.1% for the patients with a partial response (pPR) and 57.0% for the patients with a non-response (pNR).^8^ Moreover, data from the PRADO trial found that for patients with an MPR, de-escalation of both adjuvant therapy and surgery (index node excision [INE] alone rather than total lymph node dissection [TLND]) was feasible and did not impact RFS.^9,11^ The superiority of neoadjuvant therapy is clear, but the optimal perioperative strategy, including the ICI regimen and the surgical approach, remains undetermined.

Despite the prognostic utility of pathologic response, a subset of patients who experience an MPR have recurrence, and many patients who do not achieve an MPR have durable anti-tumor control. Currently, no alternative reliable prognostic or predictive biomarker is routinely applied in the clinic. Although other tissue-based biomarkers such as baseline tumor mutational burden (TMB) and tumor-infiltrating lymphocytes have been shown to prognosticate outcomes to both adjuvant and neoadjuvant ICI, the data on utility are conflicting.^11,12^ Sequential peripheral blood sampling has shown that responders develop a markedly more diverse T cell repertoire after neoadjuvant ICI than non-responders.^10^ Although promising, these time-intensive assays have yet to be incorporated into the clinic to guide patient management.

Circulating tumor DNA (ctDNA) has demonstrated promise as a predictive biomarker of ICI response in the metastatic setting and, unlike pathologic response, ctDNA can be longitudinally assessed.^13,14^ Recent data have suggested that postoperative detection of ctDNA after neoadjuvant therapy in melanoma patients is predictive of recurrence.^15^ However, the role of ctDNA as a biomarker throughout neoadjuvant immunotherapy for patients with melanoma has yet to be defined. We present real-world data examining the role of ctDNA as a biomarker to guide patient management and treatment de-escalation for patients treated with neoadjuvant immunotherapy.

## Methods

### Patient Selection

We performed a retrospective analysis of 76 patients who had high-risk resectable melanoma treated with neoadjuvant immunotherapy comprising anti-PD1 monotherapy or dual ICI between January 2020 and December 2024. Of these patients, 55% were treated with ipilimumab plus nivolumab (Ipi/Nivo), 41% were treated with anti-PD1 monotherapy, and 4% were treated with nivolumab plus relatlimab (Nivo/Rela). We did not include patients treated in clinical trials.

Most of the cohort (93%) had stage III disease with a cutaneous primary (75%). All the included patients had macroscopic disease, defined as either clinically palpable or radiologically evident, and were staged according to the American Joint Committee on Cancer (AJCC) eighth edition.^16^ Signatera ctDNA results were available and reviewed for 22 of the 76 patients.

### Data Collection

Before initiation of neoadjuvant therapy, we assessed baseline patient demographic and clinical data. Additionally, genomic data generated as part of routine clinical care using an in-house next-generation sequencing (NGS) panel was collected.^17^ After initiation of neoadjuvant therapy, data were collected on immune-related adverse events (irAEs), surgical-related AEs, pathologic response, adjuvant systemic therapy, and survival outcomes (EFS, RFS). Pathologic assessment of the resected specimen was documented by a clinical pathologist in accordance with the International Neoadjuvant Melanoma Consortium grading criteria.^18^ Patients with a pathologic complete response (pCR: no viable tumor cells present within the resected specimen) and near complete response (nearCR: 10% or less tumor viability) were defined as having a major pathologic response (MPR), and all others were defined as not having an MPR (non-MPR). Three patients were defined as mixed responses when resection of multiple distinct tissues showed discordant pathologic responses (e.g., a patient with a pCR in a lymph node and pNR in an in-transit lesion). The study defined EFS as the time from the first cycle of neoadjuvant ICI to disease progression, postoperative recurrence, or death. Clinical recurrence was detected either radiologically or clinically, and where clinically appropriate, confirmed histologically. The study defined RFS as the time from surgery to recurrence. Patients who had no recurrence were censored at the last follow-up visit.

Detection and quantification of ctDNA was performed using Signatera (Natera, Inc, Austin, TX, USA). This platform has already been well-described.^19,20^ In short, Signatera is a tumor-informed assay in which patient tumors undergo whole-exome sequencing (WES) alongside a matched sample of normal blood to generate a unique targeted panel for subsequent ctDNA detection. Where possible, plasma samples were collected for the following timepoints: during pre-neoadjuvant ICI, during neoadjuvant ICI, preoperatively, and postoperatively.

### Statistical Analysis

Continuous variables were summarized by median and interquartile range (IQR) and categorical variables by frequency and percentage. Pearson’s chi-square or Fisher’s exact test was used for statistical analysis of categorical data. The Kaplan-Meier method was used to display survival times (EFS and RFS), and statistical comparison between subgroups was performed using the log-rank test. Hazard ratios (HRs) were estimated using the Cox proportional hazards model. To investigate factors associated with an MPR, univariate logistic regression models were fitted.

All statistical analyses were performed using R software (version 4.3.2, packages: Survminer, Survival, tidymodels, and ggplot2), and significance was defined as a two-tailed *p* value lower than 0.05.

### Study Approval and Ethics

This study received institutional review board (IRB) approval, and all patients consented to the use of clinical and sample data for research purposes.

## Results

### Baseline Characteristics

**Fig. 1:**
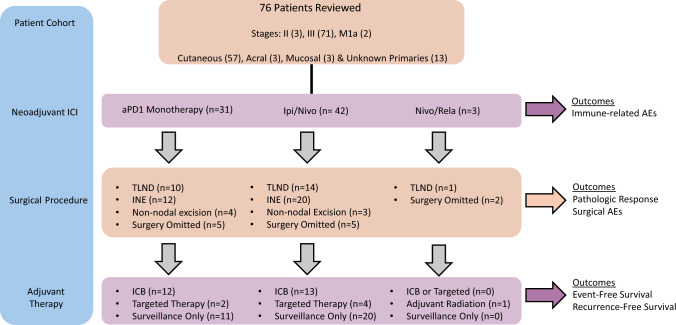
Flow chart illustrating patient selection and annotated outcomes. For this analysis, 75 patients treated with neoadjuvant intent were reviewed. The majority were treated with ipilimumab/nivolumab or aPD1 monotherapy, which included pembrolizumab (*n* = 29) and nivolumab (*n* = 2). Analysis excluded the nivolumab plus relatlimab cohort due to the small number of patients. Mucosal melanoma patients were excluded from outcome analyses due to the small cohort size and exclusion from neoadjuvant randomized clinical trials. aPD1, anti-programmed cell death protein 1; AE, adverse events; BRAFi/MEKi, BRAF/MEK inhibitor; ctDNA, circulating tumor DNA; EFS, event-free survival; ICI, immune checkpoint inhibitor; INE, index lymph node excision; Ipi, ipilimumab; Nivo, nivolumab; RFS, recurrence-free survival; TLND, total lymph node dissection

**Fig. 2:**
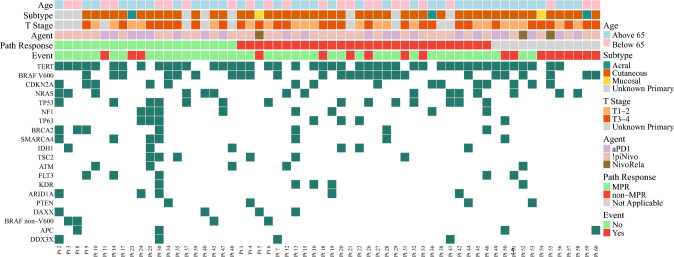
Comutation plot displaying clinical, histologic, and somatic mutation data. Each column represents a patient. Only genes mutated in at least three patients are displayed. For all rows representing genes, white spaces represent wild-type mutational status. Surgery was omitted for patient 52 due to an excellent clinical response to neoadjuvant therapy. aPD1, anti-programmed cell death protein-1; IpiNivo, ipilimumab plus nivolumab; MPR, major pathologic response; NivoRela, nivolumab plus relatlimab

The study identified 76 patients treated with neoadjuvant immunotherapy between April 2020, and November 2024 (Fig. 1).The median age at the start of neoadjuvant immunotherapy was 66 years (IQR, 60.8–77.3 years). A primary histopathologic subtype was identified in 63 patients (82.9%), in whom non-acral cutaneous melanoma was predominant (75.0%). Three patients had primary acral melanomas, and three patients had primary mucosal melanomas. Among the patients measured (*n* = 75), 69.7% had an Eastern Cooperative Oncology Group (ECOG) score of 0, and 28.9% had a score of 1 documented. In-house genetic tumor-profiling was performed for 60 (78.9%) of the patients. Targeted BRAF assessment was performed for a further four patients without genetic tumor-profiling available. For 36 patients (47.4%), BRAF mutations were diagnosed, 32 (42.1%) of which were activating V600 mutations (Fig. 2). Additional baseline characteristics are summarized in Table 1.

**Table 1:** Baseline characteristics of the study cohort

Characteristic	Ipilimumab plus nivolumab (*n* = 42) *n* (%)	aPD1 monotherapy (*n* = 31)* n* (%)	Nivolumab plus relatlimab (*n* = 3) *n* (%)	*p* Value^a^
Median age: years (IQR)	64 (50–69)	75 (64–82)	66 (49–80)	<0.001
Sex	–	–	–	–
Male	30 (71.4)	24 (77.4)	3 (100.0)	0.76
Female	12 (28.6)	7 (22.6)	0 (0.0)	–
Race	–	–	–	0.94
White	39 (92.9)	28 (90.3)	3 (100)	–
Non-white or not reported	3 (7.1)	3 (9.7)	0 (0.0)	–
Primary melanoma subtype	–	–	–	0.17
Cutaneous	28 (66.7)	27 (87.1)	2 (66.7)	–
Unknown primary	11 (26.2)	2 (6.5)	0 (0.0)	–
Acral	2 (4.7)	1 (3.2)	0 (0.0)	–
Mucosal	1 (2.3)	1 (3.2)	1 (33.3)	–
T stage	–	–	–	0.13
T1-2	10 (23.8)	16 (51.6)	1 (33.3)	–
T3-4	21 (50.0)	13 (41.9)	2 (66.7)	–
Not applicable	11 (26.2)	2 (6.5)	0 (0)	–
Ulceration	–	–	–	0.89
Present	14 (33.3)	12 (38.7)	0 (0)	–
Absent	15 (35.7)	15 (48.4)	3 (100.0)	–
Unknown	12 (28.6)	4 (12.9)	0 (0.0)	–
Clinical stage	–	–	–	1
II	2 (4.8)	1 (3.2)	0 (0.0)	–
III	39 (92.9)	29 (93.5)	3 (100.0)	–
IV	1 (2.4)	1 (3.2)	0 (0.0)	–
No. of macroscopic lesions	–	–	–	0.36
1	20 (45.2)	19 (58.1)	2 (33.3)	–
≥ 2	22 (52.4)	12 (38.7)	1 (33.3)	–
Dimensions of the largest lesions (cm)	3.7	2.4	2.1	–
ECOG	–	–	–	0.12
0	33 (78.6)	18 (58.1)	2 (66.7)	–
1	9 (21.4)	12 (38.7)	1 (33.3)	–
Not reported	0 (0)	1 (3.2)	0 (0.0)	–
Prior adjuvant BRAFi/MEKi-Yes	6 (14.3)	0 (0.0)	0 (0.0)	–
BRAF status	–	–	–	0.034
V600^b^	23 (54.8)	7 (22.6)	2 (66.7)	–
non-V600	4 (9.5)	0 (0.0)	0 (0.0)	–
Wild type	14 (33.3)	14 (45.2)	1 (33.3)	–
Not reported	1 (2.4)	10 (32.3)	0 (0.0)	–
Tumor mutational burden	–	–	–	1
Low	21 (50.0)	12 (38.7)	2 (66.7)	–
High	8 (19.0)	4 (12.9)	0 (0.0)	–
Not reported	13 (31.0)	15 (48.4)	1 (33.3)	–

IQR, interquartile range; ECOG, Eastern Cooperative Oncology Group; BRAFi/MEKi, BRAF/MEK inhibitor

^a^Statistical comparison was between ipilimumab plus nivolumab and anti-PD1 groups

^b^BRAFV600 mutations included V600E, V600R, and V600K

### Treatment and Surgery

Before receipt of neoadjuvant ICI, six (7.9%) patients received prior BRAFi/MEKi in the adjuvant setting, and the remaining patients were treatment-naive. Ipi/Nivo was the most common neoadjuvant regimen (55.3%), followed by anti-PD1 monotherapy (40.8%) and nivolumab plus relatlimab (Nivo/Rela, 3.9%). Pembrolizumab was more frequently prescribed than nivolumab monotherapy (38.2% vs 2.6%). The median number of cycles before surgery was two, with Ipi/Nivo having three cycles with anti-PD1 monotherapy. The median time to surgery was 7.7 weeks (IQR, 7.2–10.1 weeks) for Ipi/Nivo and 10.8 weeks (IQR, 8.9–12.4 weeks) for the anti-PD1 subgroups. The patients treated with Ipi/Nivo were younger (median age, 64 years; IQR, 50–69 years) than those treated with anti-PD1 (age, 75 years; IQR, 64–82 years) (*p* <0.01). Patients with a BRAF V600 mutation represented a greater proportion of patients treated with Ipi/Nivo (54.8%) than those treated with anti-PD1 (22.6%) (*p* <0.001). However, 10 patients in the anti-PD1 cohort had an unknown BRAF status at the time of treatment initiation. The patients treated with Ipi/Nivo trended toward a higher burden of disease at baseline, categorized by number of lesions and size of largest lesion at baseline, but this difference was not statistically significant. More patients (52.4%) treated with Ipi/Nivo had two or more sites of disease present at baseline than patients in the anti-PD1 cohort (38.7%) (*p* = 0.36). Statistical comparison with the Nivo/Rela cohort was not deemed appropriate given the low number of patients (*n *= 3).

After the neoadjuvant course, surgery was successfully performed for 64 patients (84.2%). Surgery was not completed for 12 patients due to disease progression on neoadjuvant ICI (*n* = 6, 7.9%), clinical complete response (*n* = 3, 3.9%), and toxicity (*n* = 2, 2.6%) (Table 2). Additionally, one patient in the Ipi/Nivo cohort received bridging neoadjuvant BRAFi/MEKi therapy before surgical resection due to progressive disease. Of the 64 patients who completed surgery, 25 (39.1%) underwent upfront total lymph node dissection (TLND), and 32 (50%%) had an index node excision (INE), with reflex TLND ultimately performed for 7 patients due to non-MPR. Seven patients (10.9%) underwent curative resection of soft tissue metastases.

**Table 2:** Summary of surgical procedure, pathologic response, and adjuvant therapy

Characteristic	Ipilimumab plus nivolumab^a^ (*n* = 42) *n* (%)		aPD1 monotherapy^a^ (*n* = 31) *n* (%)		Nivolumab plus relatlimab^a^ (*n* = 3) *n* (%)	
Median weeks to surgery (IQR)^b^	7.7 (7.3–10.1)		10.8 (8.9–12.4)		8.4	
Surgery performed (yes)	37 (88.1)		26 (83.9)		1 (33.3)	
Surgery performed (no, reason)	5 (11.9)		5 (16.1)		2 (66.7)	
Progression	3 (7.1)		2 (6.5)		1 (33.3)	
Toxicity	1 (2.4)		1 (3.2)		0 (0.0)	
Response	1 (2.4)		1 (3.2)		1 (33.3)	
Other	0 (0.0)		1 (3.2)		–	
Surgical procedure	–		–		–	
INE	13 (35.1)		12 (38.7)		0 (0.0)	
INE with reflex TLND	7 (18.9)		0 (0.0)		0 (0.0)	
Upfront TLND	14 (33.3)		10 (32.3)		1 (33.3)	
Non-nodal resection	3 (7.1)		4 (12.9)		0 (0.0)	
Pathologic response	–		–		–	
pCR	15 (35.7)		17 (54.8)		0 (0.0)	
Near pCR	3 (7.1)		0 (0.0)		0 (0.0)	
pPR	3 (7.1)		0 (0.0)		0 (0.0)	
pNR	14 (33.3)		8 (25.8)		1 (33.3)	
Mixed response^c^	2 (4.8)		1 (3.2)		0 (0.0)	
Categorized pathologic response	MPR (*n* = 18) *n* (%)	notMPR (*n* = 19) *n* (%)	MPR (*n* = 17) *n* (%)	notMPR (*n* = 9) *n* (%)	MPR (*n* = 0) *n* (%)	notMPR (*n* = 1) *n* (%)
Post-surgical Management	–	–	–	–	–	–
Adjuvant	–	–	–	–	–	–
Immunotherapy	1 (5.5)	12 (63.2)	9 (52.9)	3 (33.3)	0 (0.0)	0 (0.0)
Adjuvant BRAFi/MEKi	0 (0.0)	4 (21.1)	0 (0.0)	2 (22.2)	0 (0.0)	0 (0.0)
Adjuvant radiation	0 (0.0)	0 (0.0)	0 (0.0)	0 (0.0)	0 (0.0)	1 (100.0)
No adjuvant therapy	17 (94.4)	3 (15.8)	8 (47.0)	3 (33.3)	0 (0.0)	0 (0.0)
Unknown	0 (0.0)	0 (0.0)	0 (0.0)	1 (11.1)	0 (0.0)	0 (0.0)

aPD1, anti-programmed cell-death protein-1; IQR, interquartile range; INE, index lymph node excision; TLND, total lymph node dissection; pCR, pathologic complete response; pPR, pathologic partial response; pNR, pathologic non-response; BRAFi/MEKi, BRAF/MEK inhibitor

^a^*n* (%) totals may not sum to 100% due to rounding

^b^Weeks from cycle 1 until surgery

^c^Mixed response included patients with resection of multiple distinct tissues showing discordant pathologic responses

### Pathologic Response and Adjuvant Systemic Therapy

**Fig. 3:**
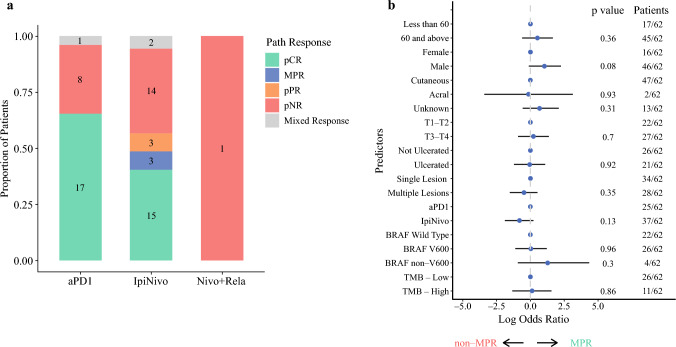
Associations with pathologic response. **A** Stacked bar chart showing the relative frequencies of pathologic response. Bars were arranged by the neoadjuvant group: aPD1 (*n* = 26) and IpiNivo (*n* = 37). Three patients were categorized as having mixed responses. For these patients, resection of multiple distinct tissues showed discordant pathologic responses. These patients were included as non-major pathologic responses. **B** Univariate analyses for major pathologic response. Forest plot represents the log odds ratio estimate of having a major pathologic response based on the predictor level. Error bars represent the 95% confidence interval. The reference level for each predictor is shown by a single point with no confidence interval. aPD1, anti-programmed cell-death protein-1; IpiNivo, ipilimumab plus nivolumab; NivoRela, nivolumab plus relatlimab; pCR, pathologic complete response; pPR, pathologic partial response; pNR, pathologic non-response; Pt, patient; TMB, tumor mutational burden

Pathologic response was evaluable for all the patients who underwent surgery (*n* = 64). In the entire cohort, MPR was documented in 35 (54.7%) of the patients, with pCR being the most common (*n* = 32). Similarly, in the 29 patients (45.3%) without MPR (non-MPR), pNR was most frequent (*n* = 21, Table 2). Univariate analyses demonstrated that no measured baseline variable significantly informed the odds ratio for achievement of an MPR (Fig. 3B). The major pathologic response rate did not significantly differ statistically between the anti-PD1 and Ipi/Nivo groups, with rates of 65.4% and 48.6% respectively (*p* = 0.2; Fig. 3A). Moreover, comparative analyses between tumor mutational burden and pathologic response demonstrated no significant associations (Table S1).

Among the 64 patients who completed surgery, 31 (48.4%) received adjuvant systemic therapy. Of these patients, 25 continued with immunotherapy (anti-PD1), and 6 received BRAFi/MEKi. Use of adjuvant systemic therapy was significantly higher in the non-MPR cohort than in the MPR cohort (72.4% vs 28.6%; *p* < 0.01). For 6 of the 29 patients without MPR, adjuvant therapy was deferred, 2 due to toxicity and 4 due to patient/provider preference. Three of the six non-MPR patients who did not receive adjuvant therapy had recurrence.

### ctDNA Detection and Neoadjuvant Response

Of the 64 patients who underwent surgery, 22 (34.4%) had plasma collected both pre- and postoperatively for ctDNA analysis. Before ICI initiation, 19 of these patients had ctDNA results available. Demographics, treatment, response rates and recurrence rates were similar between the patients with serial ctDNA and those without ctDNA. The patients with ctDNA had lower ECOG scores and higher TMB than the patients without ctDNA.

**Fig. 4:**
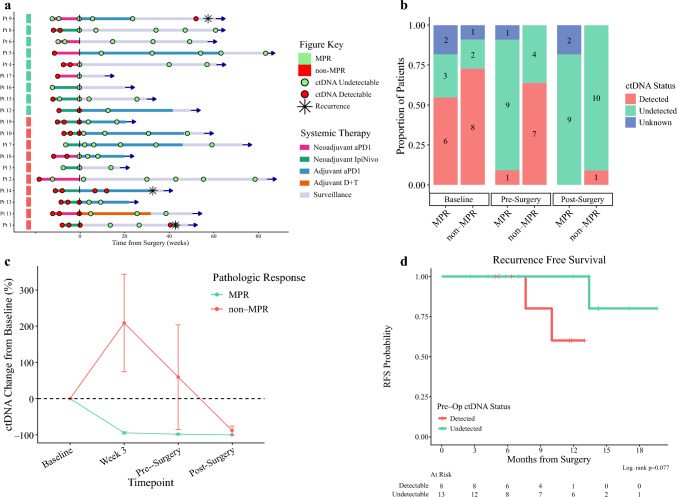
Undetected ctDNA corresponds with favorable clinical outcomes. **A** The swimmer plot illustrates the clinical timeline and sample collection for ctDNA analysis. Each row represents a unique patient, and rows were ordered by pathologic response. The vertical line corresponds with the day of surgery (time, 0 weeks). Patients with missing baseline ctDNA data were not illustrated. **B** Patients with both pre- and postoperative ctDNA (*n * = 22) were grouped by pathologic response and sample timepoint (baseline, preoperative, and postoperative). This bar chart represents the proportion of patients with detectable or undetectable ctDNA at each time point. If ctDNA was not tested, data were documented as unknown. **C** Line plot demonstrating percentage change (mean ± SE) in ctDNA relative to the baseline value over time. Patients were grouped by pathologic response. Given that this was a relative change, patients with undetectable ctDNA at baseline (0 MTM/ml) or unknown data at baseline were excluded (*n* = 10, total). **D** RFS stratified by preoperative ctDNA status (*n* = 21). ctDNA, circulating tumor DNA; SE, standard error; ICI, immune checkpoint inhibitor; MPR, major pathologic response; MTM, mean tumor molecules; RFS, recurrence-free survival

The baseline characteristics of the ctDNA cohort are summarized in Table S3. Of the 19 patients with evaluable ctDNA before ICI, 14 (73.7%) had detectable ctDNA (Fig. 4A). Six patients cleared ctDNA by the time of surgery (zero-converted), and none of these patients had recurrence. Despite neoadjuvant immunotherapy, ctDNA was detected in eight patients preoperatively, two of whom had recurrence. One of the five patients without detected ctDNA at any preoperative timepoint had recurrence (Table S2). At baseline, no statistical difference was observed in rates of ctDNA detection between the MPR and non-MPR cohorts (*p* = 0.6). However, pre-surgery analysis showed significantly lower rates of ctDNA detection in the MPR cohort (10.0%) than in the non-MPR cohort (63.6%) (*p* = 0.038). This observed difference was not evident after analysis of the earliest postoperative sample, in which all but one patient was undetectable (Fig. 4B). Two patients in this cohort had a mixed pathologic response. One patient had pNR with 50% viable tumor in the lymph node and pCR in an in-transit lesion. The second patient had pCR in the lymph node, but 85% viable tumor in an in-transit lesion. In both cases ctDNA was undetectable at the preoperative timepoint.

Quantitative analysis of ctDNA showed notable variation in the non-MPR group at week 3 and preoperatively. Ten patients had detectable ctDNA and a known value at week 3. Four of the six patients with a decrease in ctDNA between baseline and week 3 were undetectable immediately before surgery. None of the six patients had recurrence. On the other hand, 100% (4/4) of the patients with an increase in ctDNA at week 3 had detectable ctDNA immediately before surgery. All of these patients were pNR. To date, 50% (2/4) have had recurrence. This highlights the potential ability of ctDNA to detect evidence of resistance, and ultimately non-MPR early. At the first postoperative blood draw, only one patient had detected levels, and this patient subsequently had recurrence (Fig. 4C). Moreover, all postoperative recurrences were preceded by detected levels of ctDNA (Fig. 4A)*.* Only one patient in this ctDNA cohort received adjuvant BRAF/MEKi, and this patient has not experienced recurrence.

Finally, we analyzed RFS stratified by preoperative ctDNA status (*n* = 21). At 12-months, the RFS rate was 100.0% (95% CI not applicable) for the patients with undetected levels of ctDNA and 60.0% (95% CI, 0.29–100%) for the patients with detected levels of ctDNA (Fig. 4D).

### Safety

*irAEs.* Among 45 patients (59.2%) who experienced immune-related adverse events (irAEs) of any grade, 26 (61.9%) received Ipi/Nivo, and 17 (54.8%) received anti-PD1. Two of the three patients treated with Nivo/Rela experienced toxicity. Of 27 (35.5%) patients who experienced irAEs requiring treatment with steroids, 17 were in the Ipi/Nivo cohort, and 10 were in the anti-PD1 cohort. Notably, four patients experienced myocarditis (2 receiving anti-PD1, 2 receiving Ipi/Nivo), including one grade 5 event after one cycle of Ipi/Nivo. The Ipi/Nivo cohort had significantly more endocrine toxicities (*p* = 0.023), including two cases of ICI-related diabetes mellitus. Additional data are summarized in Table S4.

### Surgical Morbidity

The adverse surgical outcomes for the 64 patients who underwent 71 surgical procedures are summarized in Table S5. Postoperative drain use was more frequent after TLND than after INE (93.8% vs 18.8%; *p* < 0.01). Wound infection requiring intravenous antibiotics were uncommon and reported only in the TLND subgroup (*n* = 4; 12.5%).

### Survival Outcomes

**Fig. 5:**
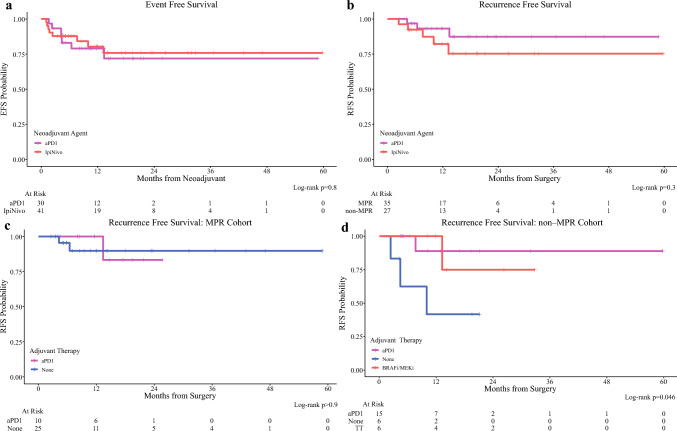
Survival outcomes. **A** EFS for all patients who received at least one dose of neoadjuvant ICI (*n* = 71). Patients with a diagnosis of mucosal melanoma and those treated with neoadjuvant nivolumab plus relatlimab (*n* = 5, total) were excluded. **B** RFS for patients (*n* = 62) who completed surgery by pathologic response subgroup. Patients with a diagnosis of mucosal melanoma and those treated with neoadjuvant nivolumab plus relatlimab (*n *= 2 total) were excluded. **C** RFS for patients with a MPR (*n* = 35), grouped by subsequent adjuvant therapy. **D** RFS for patients without a MPR (*n* = 27) stratified by subsequent adjuvant therapy. The median RFS was 10 months for patients who received no adjuvant therapy and not reached for those who received adjuvant therapy. EFS, event free survival; ICI, immune checkpoint inhibitor; RFS, recurrence-free survival; MPR, major pathologic response

At data cutoff, the median follow-up period was 15.8 months (IQR, 9.8–23.5 months). The patients with mucosal melanoma and those treated with Nivo/Rela were excluded from survival analyses given the unique biologic phenotype and low numbers, respectively. During the follow-up period, 15 patients had an event, 8 of which were postoperative disease recurrence. A further six patients who did not complete surgery had disease progression, and one patient had neoadjuvant therapy that resulted in a grade 5 event. Among the 71 evaluable patients, the 12-month EFS was 79.8% (95% CI, 70.4–90.4%) and the RFS was 88.1% (95% CI, 79.5–97.7%). No difference was observed between the 12-month EFS of the Ipi/Nivo and anti-PD1 groups, with rates of 80.3% and 79.0% respectively (Fig. 5A).

Among the 64 patients who underwent surgery, 62 were included for RFS estimation. Eight patients experienced disease recurrence (3 patients from the MPR subgroup and 5 patients from the non-MPR subgroup). One patient with a mucosal primary who received nivolumab plus relatlimab also had recurrence but was excluded from RFS estimations due to subtype and immunotherapy agent. At 12 months, those with an MPR demonstrated a better RFS (93.1%; CI, 84.4–100%) than those in the non-MPR cohort (82.2%; CI, 67.5–100%). This pattern was similar at the 24-month follow-up evaluation, with rates of 87.3% (CI, 77.4–100%) and 75.3% (CI, 58.1–97.7%), respectively (*p* = 0.3; Fig. 5B). For those with an MPR, subsequent adjuvant systemic therapy had no significant influence on RFS (Fig. 5C). Yet, for the patients without an MPR, adjuvant therapy was associated with an improved RFS (93.3%, vs 41.7%; *p* = 0.014) at the 12-month follow-up evaluation (Fig. 5D).

## Discussion

Neoadjuvant ICI has become the standard of care in the macroscopic, resectable setting, but few studies have characterized the use of neoadjuvant therapy in clinical practice. From this retrospective, single-institution study, we present data from patients treated with either combination or single-agent neoadjuvant ICI. In a real-world setting, we observed survival outcomes comparable with those from prospective randomized studies. Furthermore, we found that ctDNA results may complement pathologic response in prognosticating risk of recurrence and identifying patients who have benefited from neoadjuvant immunotherapy.

Neoadjuvant ICI was generally well-tolerated in our cohort, with expected rates of toxicity (irAE and surgical AEs). Steroid use to address irAEs was comparable between combination and single-agent therapies. The Ipi/Nivo cohort had one fatal case of myocarditis. Disease progression and this treatment-related death prevented surgical care for a total of six patients. Notably, three additional patients opted out of surgery after an excellent clinical response to neoadjuvant ICI, and to date have remained disease-free. Parallels are noted with the SWOG-1801 trial, whereby one patient with complete clinical response declined surgery and at the time of publication was disease-free.^8^ Complete omission of surgery based on clinical and radiologic response has not been prospectively studied. In the PRADO trial, patients with an MPR after neoadjuvant Ipi/Nivo underwent an INE only. In our real-world practice, clinicians extrapolated and applied this de-escalation approach to patients treated with neoadjuvant anti-PD1 as well as dual ICI. We observed this approach to be safe, with comparable rates of recurrence between INE (9.4%) and TLND (12.0%). A reflex TLND was ultimately performed for 7 of the 11 patients with non-MPR after INE, in keeping with the study design of PRADO. Four patients without an MPR did not have reflex TLND, one because of disease location outside a basin, one because of comorbidities, and two at the discretion of the treating team. As expected, use of a drain postoperatively and lymphedema were higher after TLND than after INE. This trend was consistent with that seen in the MSLT-2 trial.^21^

No phase 3 randomized controlled trial (RCT) has directly compared single-agent with combination therapy in the neoadjuvant setting, so the optimal ICI regimen remains undetermined. Our data show that the patients treated with Ipi/Nivo and anti-PD1 had pathologic response rates of 48.6% and 65.4% respectively, which differ from previously published data, including work by Amaria et al., ^10^ in which the response rates were 73% for the Ipi/Nivo and 25% for the nivolumab-only arms of the study. We hypothesize that selection bias likely influenced these results in our cohort because the patients treated with Ipi/Nivo tended to have a higher burden of disease and were more likely to have a BRAF mutation.

At 12-months, we observed an EFS of 80.3% in the Ipi/Nivo cohort and 79.0% in the anti-PD1 cohort. The estimated 12-month EFS in the neoadjuvant arm of the NADINA trial was 83.7%, whereas it was estimated to be 72% in the neoadjuvant arm of SWOG-1801.^7,8^ Comparison of RFS between Ipi/Nivo and anti-PD1 showed no statistical difference in our cohort. However, analysis of pooled prospective trial data showed a statistically significant increase in RFS at 12-months for the patients receiving Ipi/Nivo (84%) compared with those receiving anti-PD1 (64%).^22^ Extrapolation of these results is limited by the heterogeneity of study designs and the low number of patients, especially in the anti-PD1 group (*n* = 37).

We found that our clinicians adopted the NADINA trial approach of response-directed de-escalation of adjuvant therapy irrespective of neoadjuvant regimen. In our dataset, this extrapolation to neoadjuvant anti-PD1 did not impact outcomes, although the cohort size was not powered to see a difference. For the patients with MPR, subsequent adjuvant therapy did not influence RFS. However, adjuvant systemic therapy significantly improved RFS among the patients who did not achieve MPR. These findings were concordant with those of another retrospective study that demonstrated an improved RFS with adjuvant therapy for patients with a pNR (*n* = 35).^23^ In the PRADO study, patients with a pNR received adjuvant therapy, whereas those with a pPR did not. At 12 and 24-months, RFS was higher in the pNR subgroup, a difference potentially attributable to the administration of adjuvant therapy.^24^ Conversely, in an aggregated cohort of 49 patients with a pNR, adjuvant ICI did not significantly improve survival outcomes.^22^ The role of adjuvant ICI for patients without a major pathologic response remains a subject of debate, and identifying factors for further stratification of this subgroup would be of considerable clinical value.

Pathologic response is a useful biomarker but cannot perfectly predict long-term outcomes.^25^ Therefore, we evaluated the utility of ctDNA as a complementary biomarker in patients treated with neoadjuvant immunotherapy. Commercially, ctDNA testing is available, and the minimally invasive nature of plasma collection allows for longitudinal analysis. Dynamic reductions in ctDNA have been associated with a favorable response to ICI in the metastatic setting.^26^ Studies have successfully used ctDNA to guide treatment decisions in other malignancies.^27,28^ We reported similar rates of ctDNA detection at baseline between the patients who had a MPR and those who did not. Interestingly, 3 weeks after cycle 1 of neoadjuvant ICI, we observed a marked reduction in the ctDNA levels of patients who achieved an MPR compared with those who did not. At the first postoperative blood draw, ctDNA was undetected in all but one patient, who had recurrence. This is consistent with a study of patients treated with neoadjuvant Ipi/Nivo, in which all the patients with detectable ctDNA experienced postoperative recurrence and consequently had a significantly shorter RFS.^15^ The vast majority of patients have undetectable ctDNA postoperatively, so we focused on the pre-surgical ctDNA dynamics that may be informative for a greater number of patients. We found that irrespective of baseline ctDNA, detection of ctDNA before surgery was associated with a shorter RFS. Moreover, none of the patients with detectable levels of ctDNA at baseline that became undetectable before surgery experienced recurrence. This was consistent with findings from Chan et al.^15^ showing that no patient who was ctDNA-positive at baseline and subsequently cleared (*n* = 17) relapsed postoperatively.^15^ This on-treatment reduction in ctDNA and the associated more favorable clinical outcome may point to a predictive capacity of ctDNA.

This study had several limitations. First, it was a single-institution retrospective study and therefore subject to selection bias. The sample size of the ctDNA cohort was not powered for statistical analyses. This was also true of analysis within our defined pathologic subgroups. However, few retrospective cohort studies have a larger sample size for analysis, and even fewer have integrated longitudinal monitoring of ctDNA. On the average (median), each patient had five samples collected, which covered key clinical timepoints (baseline, on-treatment, preoperatively, and postoperatively), thus giving greater depth to clinical correlations.

In summary, we assessed clinical outcomes after neoadjuvant ICI in a real-world cohort and observed that the reported survival outcomes from prospective trials are generalizable to patients treated in the clinic. Furthermore, response-directed de-escalation in surgical and medical treatments was safe without compromise to survival outcomes. Adjuvant systemic therapy for patients without an MPR was associated with reduced rates of recurrence and an increased RFS. Furthermore, serial assessment of ctDNA may provide earlier read-outs of biologic response and may complement pathologic response in guiding patient management. These data are exploratory and require validation in prospective studies with larger cohorts.

## Supplementary Information

Below is the link to the electronic supplementary material.

Supplementary file1 (DOCX 30 KB)

## References

[1] Zhang S, Bensimon AG, Xu R, et al. Cost-effectiveness analysis of pembrolizumab as an adjuvant treatment of resected stage IIB or IIC melanoma in the United States. *Adv Ther*. 2023;40:3038–55. 10.1007/s12325-023-02525-x. 37191852 10.1007/s12325-023-02525-x PMC10271902

[2] Eggermont AMM, Blank CU, Mandala M, et al. Longer follow-up confirms recurrence-free survival benefit of adjuvant pembrolizumab in high-risk stage III melanoma: updated results from the EORTC 1325-MG/KEYNOTE-054 trial. *J Clin Oncol*. 2020;38:3925–36. 10.1200/JCO.20.02110. 32946353 10.1200/JCO.20.02110 PMC7676886

[3] Ascierto PA, Del Vecchio M, Mandalá M, et al. Adjuvant nivolumab versus ipilimumab in resected stage IIIB-C and stage IV melanoma (CheckMate 238): 4-year results from a multicentre, double-blind, randomised, controlled, phase 3 trial. *Lancet Oncol*. 2020;21:1465–77. 10.1016/S1470-2045(20)30494-0. 32961119 10.1016/S1470-2045(20)30494-0

[4] Long GV, Hauschild A, Santinami M, et al. Final results for adjuvant dabrafenib plus trametinib in stage III melanoma. *N Engl Jo Med*. 2024;391(18):1709–20. 10.1056/NEJMoa2404139. 10.1056/NEJMoa2404139 38899716

[5] Mohr P, Kiecker F, Soriano V, et al. Adjuvant therapy versus watch-and-wait post-surgery for stage III melanoma: a multicountry retrospective chart review. *Melanoma Manag*. 2019. 10.2217/mmt-2019-0015. 31871622 10.2217/mmt-2019-0015 PMC6923782

[6] Tonella L, Pala V, Ponti R, et al. Prognostic and predictive biomarkers in stage III melanoma: current insights and clinical implications. *Int J Mol Sci*. 2021;22:4561. 10.3390/ijms22094561. 33925387 10.3390/ijms22094561 PMC8123895

[7] Blank CU, Lucas MW, Scolyer RA, et al. Neoadjuvant Nivolumab and Ipilimumab in resectable stage III melanoma. *N Engl J Med*. 2024;391:1696–708. 10.1056/NEJMoa2402604. 38828984 10.1056/NEJMoa2402604

[8] Patel SP, Othus M, Chen Y, et al. Neoadjuvant–adjuvant or adjuvant-only pembrolizumab in advanced melanoma. *N Engl J Med*. 2023;388:813–23. 10.1056/NEJMoa2211437. 36856617 10.1056/NEJMoa2211437 PMC10410527

[9] Amaral T, Ottaviano M, Arance A, et al. Cutaneous melanoma: ESMO clinical practice guideline for diagnosis, treatment, and follow-up☆. *Ann Oncol*. 2025;36:10–30. 10.1016/j.annonc.2024.11.006. 39550033 10.1016/j.annonc.2024.11.006 PMC7618628

[10] Amaria RN, Reddy SM, Tawbi HA, et al. Neoadjuvant immune checkpoint blockade in high-risk resectable melanoma. *Nat Med*. 2018;24:1649–54. 10.1038/s41591-018-0197-1. 30297909 10.1038/s41591-018-0197-1 PMC6481682

[11] Larkin J, Del Vecchio M, Mandalá M, et al. Adjuvant nivolumab versus ipilimumab in resected stage III/IV melanoma: 5-year efficacy and biomarker results from CheckMate 238. *Clin Cancer Res*. 2023;29:3352–61. 10.1158/1078-0432.CCR-22-3145. 37058595 10.1158/1078-0432.CCR-22-3145 PMC10472092

[12] Rozeman EA, Hoefsmit EP, Reijers ILM, et al. Survival and biomarker analyses from the OpACIN-neo and OpACIN neoadjuvant immunotherapy trials in stage III melanoma. *Nat Med*. 2021;27:256–63. 10.1038/s41591-020-01211-7. 33558721 10.1038/s41591-020-01211-7

[13] Pascual J, Attard G, Bidard FC, et al. ESMO recommendations on the use of circulating tumour DNA assays for patients with cancer: a report from the ESMO Precision Medicine Working Group. *Ann Oncol*. 2022;33:750–68. 10.1016/j.annonc.2022.05.520. 35809752 10.1016/j.annonc.2022.05.520

[14] LaPelusa M, Qiao W, Iorgulescu B, et al. Long-term efficacy of pembrolizumab and the clinical utility of ctDNA in locally advanced dMMR/MSI-H solid tumors. *Nat Commun*. 2025;16:4514. 10.1038/s41467-025-59615-3. 40374594 10.1038/s41467-025-59615-3 PMC12081696

[15] Chan WY, Lee JH, Stewart A, et al. Circulating tumour DNA dynamics predict recurrence in stage III melanoma patients receiving neoadjuvant immunotherapy. *J Exp Clin Cancer Res*. 2024;43:1–9. 10.1186/s13046-024-03153-1. 39169411 10.1186/s13046-024-03153-1 PMC11337884

[16] Gershenwald JE, Scolyer RA, Hess KR, et al. Melanoma staging: evidence-based changes in the American Joint Committee on Cancer eighth edition cancer staging manual. *CA Cancer J Clin*. 2017;67:472–492. 10.3322/caac.21409. 10.3322/caac.21409 PMC5978683 29028110

[17] O’Donnell E, Mahindra A, Yee AJ, et al. Clinical grade “SNaPshot” genetic mutation profiling in multiple myeloma. *EBioMedicine*. 2014;2:71–3. 10.1016/j.ebiom.2014.11.008. 26137536 10.1016/j.ebiom.2014.11.008 PMC4485483

[18] Tetzlaff MT, Messina JL, Stein JE, et al. Pathological assessment of resection specimens after neoadjuvant therapy for metastatic melanoma. *Ann Oncol*. 2018;29:1861–8. 10.1093/annonc/mdy226. 29945191 10.1093/annonc/mdy226 PMC6096739

[19] Coombes RC, Page K, Salari R, et al. Personalized detection of circulating tumor DNA antedates breast cancer metastatic recurrence. *Clin Cancer Res*. 2019;25:4255–63. 10.1158/1078-0432.CCR-18-3663. 30992300 10.1158/1078-0432.CCR-18-3663

[20] Reinert T, Henriksen TV, Christensen E, et al. Analysis of plasma cell-free DNA by ultradeep sequencing in patients with stages I to III colorectal cancer. *JAMA Oncol*. 2019;5:1124–31. 10.1001/jamaoncol.2019.0528. 31070691 10.1001/jamaoncol.2019.0528 PMC6512280

[21] Faries MB, Thompson JF, Cochran AJ, et al. Completion dissection or observation for sentinel-node metastasis in melanoma. *N Engl J Med*. 2017;376:2211–22. 10.1056/NEJMoa1613210. 28591523 10.1056/NEJMoa1613210 PMC5548388

[22] Menzies AM, Amaria RN, Rozeman EA, et al. Pathological response and survival with neoadjuvant therapy in melanoma: a pooled analysis from the International Neoadjuvant Melanoma Consortium (INMC). *Nat Med*. 2021;27:301–9. 10.1038/s41591-020-01188-3. 33558722 10.1038/s41591-020-01188-3

[23] Nelson A, Krabbe E, Björkström K, et al. Neoadjuvant immunotherapy for patients with resectable stage III/IV cutaneous melanoma: a Swedish retrospective real-world study (NEO-MEL). *Eur J Cancer*. 2025. 10.1016/j.ejca.2025.115485. 40456667 10.1016/j.ejca.2025.115485

[24] Reijers ILM, Menzies AM, van Akkooi ACJ, et al. Personalized response-directed surgery and adjuvant therapy after neoadjuvant ipilimumab and nivolumab in high-risk stage III melanoma: the PRADO trial. *Nat Med*. 2022;28:1178–88. 10.1038/s41591-022-01851-x. 35661157 10.1038/s41591-022-01851-x

[25] Sharon CE, Tortorello GN, Ma KL, et al. Long-term outcomes to neoadjuvant pembrolizumab based on pathological response for patients with resectable stage III/IV cutaneous melanoma☆. *Ann Oncol*. 2023;34:806–12. 10.1016/j.annonc.2023.06.006. 37414215 10.1016/j.annonc.2023.06.006 PMC11232562

[26] Lee JH, Long GV, Boyd S, et al. Circulating tumour DNA predicts response to anti-PD1 antibodies in metastatic melanoma. *Ann Oncol*. 2017;28:1130–6. 10.1093/annonc/mdx026. 28327969 10.1093/annonc/mdx026

[27] Tie J, Wang Y, Lo SN, et al. Circulating tumor DNA analysis guiding adjuvant therapy in stage II colon cancer: 5-year outcomes of the randomized DYNAMIC trial. *Nat Med*. 2025;31:1509–18. 10.1038/s41591-025-03579-w. 40055522 10.1038/s41591-025-03579-w PMC12974608

[28] Zhou J, Huang J, Zhou Z, et al. Value of ctDNA in surveillance of adjuvant chemosensitivity and regimen adjustment in stage III colon cancer: a protocol for phase II multicentre randomised controlled trial (REVISE trial). *BMJ Open*. 2025;15:e090394. 10.1136/bmjopen-2024-090394. 39753246 10.1136/bmjopen-2024-090394 PMC11749494

