# Supplementary material for: Personalization of Neoadjuvant Immunotherapy in High-Risk Resectable Melanoma and Utility of ctDNA as a Biomarker of Immunotherapy Response
Source: Ann Surg Oncol. 2026 May 23;33(8):7449–60. doi: 10.1245/s10434-026-19814-8 (PMC13337624; doi:10.1245/s10434-026-19814-8)
Supplement: Supplementary file 1 — Supplementary file1 (DOCX 30 KB) [file 10434_2026_19814_MOESM1_ESM.docx]

***Supplemental Tables***

| **Supplemental Table 1:**  **Univariate Logistic Regression analyses of baseline somatic mutations as predictors of pathologic response**  Reference Level: non-MPR | | | | | | |
| --- | --- | --- | --- | --- | --- | --- |
| **Gene** | **MPR** | **non-MPR** | **Odd Ratio** | **CI (Lower Bound)** | **CI (Upper Bound)** | **unadjusted p value** |
| SMARCA4 | 3 | 2 | 2.05 | 0.31 | 16.76 | 0.45 |
| BRCA2 | 4 | 3 | 1.85 | 0.37 | 10.39 | 0.46 |
| NRAS | 6 | 6 | 1.38 | 0.37 | 5.18 | 0.63 |
| TSC2 | 3 | 3 | 1.32 | 0.22 | 7.82 | 0.75 |
| IDH1 | 3 | 3 | 1.32 | 0.22 | 7.82 | 0.75 |
| TP63 | 3 | 3 | 1.32 | 0.22 | 7.82 | 0.75 |
| DAXX | 2 | 2 | 1.30 | 0.15 | 11.61 | 0.80 |
| DDX3X | 2 | 2 | 1.30 | 0.15 | 11.61 | 0.80 |
| ARID1A | 2 | 2 | 1.30 | 0.15 | 11.61 | 0.80 |
| MAP2K1 | 2 | 2 | 1.30 | 0.15 | 11.61 | 0.80 |
| TP53 | 7 | 8 | 1.17 | 0.34 | 3.97 | 0.80 |
| CDKN2A | 6 | 7 | 1.12 | 0.31 | 4.04 | 0.86 |
| TERT | 17 | 22 | 0.93 | 0.24 | 3.71 | 0.91 |
| BRAF | 12 | 15 | 1.04 | 0.34 | 3.23 | 0.95 |
| NF1 | 4 | 5 | 1.02 | 0.22 | 4.41 | 0.98 |
| *Abbreviations: CI, confidence interval; MPR, major pathologic response* | | | | | | |

| **Supplemental Table 2: ctDNA status, pathologic response and recurrence status following neoadjuvant ICI**​ | | | | | | |
| --- | --- | --- | --- | --- | --- | --- |
| **ctDNA Status**​ | | | **Pathologic Response**​ | | ​ | **Clinical Outcome**​ |
| **Baseline**​ | **Pre-Surgery**​ | **Post-Surgery^a^**​ | **MPR (N=11)**​ | **non-MPR (n=11)**​ | ​ | **Recurrence / Total**​ |
| Undetected​ | Undetected​ | Undetected​ | 2​ | 2​ | ​ | 1 / 4​ |
| Undetected​ | Undetected​ | Not Tested​ | 1​ | 0​ | ​ | 0 / 1​ |
| Detected​ | Undetected​ | Undetected​ | 3​ | 2​ | ​ | 0 / 5​ |
| Detected​ | Undetected​ | Not Tested​ | 1​ | 0​ | ​ | 0 / 1​ |
| Detected​ | Not Tested​ | Undetected​ | 1​ | 0​ | ​ | 0 / 1​ |
| Detected​ | Detected​ | Undetected​ | 1​ | 5​ | ​ | 1 / 6​ |
| Detected​ | Detected​ | Detected​ | 0​ | 1​ | ​ | 1 / 1​ |
| Not Tested​ | Undetected​ | Undetected​ | 2​ | 0​ | ​ | 0 / 2​ |
| Not Tested​ | Detected​ | Undetected​ | 0​ | 1​ | ​ | 0 / 1​ |
| ^a^ References the first post-operative data only.​  *Abbreviations: Abbreviations: ctDNA, circulating tumor DNA; MPR, major pathologic response; ICI, immune checkpoint inhibitor.*​ | | | | | | |

| **Supplemental Table 3: ctDNA cohort baseline characteristics** | | | |
| --- | --- | --- | --- |
| **Characteristic** | **ctDNA Results Available (n=22)** | **No ctDNA results (n=42)** |  |
| **Age** - Median (Range) | 60 (23-82) | 66 (21 -89) |  |
| **Sex** |  |  |  |
| Male | 14 (63.6) | 33 (78.6) |  |
| Female | 8 (36.4) | 9 (21.4) |  |
| **Race** |  |  |  |
| White | 20 (90.9) | 40 (95.2) |  |
| Non-White or Not reported | 2 (9.1) | 2 (4.8) |  |
| **Primary Melanoma Subtype** |  |  |  |
| Cutaneous | 18 (81.8) | 30 (71.4) |  |
| Unknown Primary | 4 (18.2) | 8 (19.0) |  |
| Acral | 0 (0) | 2 (4.8) |  |
| Mucosal | 0 (0) | 2 (4.8) |  |
| **T Stage** |  |  |  |
| T1-2 | 7 (31.8) | 15 (35.7) |  |
| T3-4 | 10 (45.5) | 19 (45.2) |  |
| Not Applicable | 5 (22.7) | 8 (19.0) |  |
| **Ulceration** |  |  |  |
| Present | 8 (36.4) | 14 (33.3) |  |
| Absent | 8 (36.4) | 19 (45.2) |  |
| Unknown | 6 (27.2) | 9 (21.4) |  |
| **Clinical Stage** |  |  |  |
| II | 1 (4.5) | 0 (0) |  |
| III | 20 (90.9) | 41 (97.6) |  |
| IV | 1 (4.5) | 1 (2.4) |  |
| **Number of macroscopic lesions** |  |  |  |
| One | 10 (45.5) | 24 (75) |  |
| Two or more | 12 (54.5) | 18 (25) |  |
| **Dimensions of the largest lesions (cm)** | 3.6cm | 3.0cm |  |
| **ECOG** |  |  |  |
| 0 | 19 (86.4) | 25 (59.5) |  |
| 1 | 3 (13.6) | 16 (38.1) |  |
| Not Reported | 0 (0) | 1 (2.4) |  |
| **Prior adjuvant BRAFi/MEKi - Yes** | 2 (9.1) | 2 (4.8) |  |
| **BRAF Status** |  |  |  |
| V600^b^ | 8 (36.4) | 19 (45.2) |  |
| non-V600 | 0 (0) | 4 (9.5) |  |
| Wild Type | 11 (50.0) | 12 (28.6) |  |
| Not Reported | 3 (13.6) | 7 (16.7) |  |
| **Tumor Mutational Burden** |  |  |  |
| Low | 7 (31.8) | 19 (45.2) |  |
| High | 9 (40.9) | 4 (9.5) |  |
| Not Reported | 6 (27.3) | 19 (45.2) |  |
| **Immunotherapy Agent** |  |  |  |
| Ipilimumab + Nivolumab | 14 (63.6) | 23 (54.8) |  |
| aPD1 monotherapy | 8 (36.4) | 18 (42.9) |  |
| Nivolumab + Relatlimab | 0 (0) | 1 (2.4) |  |
| **Path Response** |  |  |  |
| pCR | 11 (50.0) | 21 (50.0) |  |
| near-pCR | 0 (0) | 3 (7.1) |  |
| pPR | 2 (9.1) | 1 (2.4) |  |
| pNR | 7 (31.8) | 16 (38.1) |  |
| Mixed | 2 (9.1) | 1 (2.4) |  |
| **Recurrence** |  |  |  |
| Yes | 3 (13.6) | 6 (14.3) |  |
| No | 19 (86.4) | 36 (85.7) |  |
| ^a^ N (%) |  |  |  |
| ^b^BRAF V600 Mutations included V600E, V600R and V600K. |  |  |  |

| **Supplemental Table 4: Treatment-Related Adverse Events**​ | | | | ​ |
| --- | --- | --- | --- | --- |
| **Any Grade Adverse Event**​ | **Ipilimumab plus  Nivolumab^a^**​  **N = 42**​ | **anti-PD1^a^**​  **N = 31**​ | **Nivolumab plus  Relatlimab^a^**​  **N = 3**​ | **p value^b^**​ |
| **Any Grade Adverse Event**​ | ​ | ​ | ​ | ​ |
| *Cardiac*​ | ​ | ​ | ​ | 1​ |
| Yes​ | 2 (4.8)​ | 1 (3.2)​ | 0 (0.0)​ | ​ |
| No​ | 40 (95.2)​ | 30 (96.8)​ | 3 (100.0)​ | ​ |
| *Endocrine*​ | ​ | ​ | ​ | 0.023​ |
| Yes​ | 13 (31.0)​ | 2 (6.5)​ | 1 (33.3)​ | ​ |
| No​ | 29 (69.0)​ | 29 (93.5)​ | 2 (66.7)​ | ​ |
| *Gastrointestinal*​ | ​ | ​ | ​ | 0.79​ |
| Yes​ | 9 (21.4)​ | 5 (16.1)​ | 1 (33.3)​ | ​ |
| No​ | 33 (78.6)​ | 26 (83.9)​ | 2 (66.7)​ | ​ |
| *Hepatic*​ | ​ | ​ | ​ | 0.50​ |
| Yes​ | 6 (14.3)​ | 2 (6.5)​ | 0 (0.0)​ | ​ |
| No​ | 36 (85.7)​ | 29 (93.5)​ | 3 (100.0)​ | ​ |
| *Musculoskeletal*​ | ​ | ​ | ​ | 0.67​ |
| Yes​ | 3 (7.1)​ | 4 (12.9)​ | 0 (0.0)​ | ​ |
| No​ | 39 (92.9)​ | 27 (87.1)​ | 3 (100.0)​ | ​ |
| *Skin*​ | ​ | ​ | ​ | 0.61​ |
| Yes​ | 7 (16.7)​ | 3 (9.7)​ | 0 (0.0)​ | ​ |
| No​ | 35 (83.3)​ | 28 (90.3)​ | 3 (100)​ | ​ |
| *Other^c^*​ | ​ | ​ | ​ | ​ |
| Yes​ | 3 (7.1)​ | 6 (19.4)​ | 0 (0.0)​ | 0.23​ |
| No​ | 39 (92.9)​ | 25 (80.6)​ | 3 (100.0)​ | ​ |
| **Management of Adverse Event**​ | ​ | ​ | ​ | ​ |
| *Steroids*​ | ​ | ​ | ​ | ​ |
| Yes​ | 17 (40.5)​ | 10 (32.3)​ | 2 (66.7)​ | 0.79​ |
| No​ | 25 (59.5)​ | 21 (67.7)​ | 1 (33.3)​ | ​ |
| *Mycophenolate*​ | ​ | ​ | ​ | ​ |
| Yes​ | 3 (7.1)​ | 3 (9.7)​ | 0 (0.0)​ | 1​ |
| No​ | 39 (92.9)​ | 28 (90.3)​ | 3 (100.0)​ | ​ |
| *Infliximab*​ | ​ | ​ | ​ | ​ |
| Yes​ | 3 (7.1)​ | 0 (0)​ | 1 (33.3)​ | 0.36​ |
| No​ | 39 (92.9)​ | 31 (100)​ | 2 (66.7)​ | ​ |
| *Hormone Replacement*​ | ​ | ​ | ​ | 0.12​ |
| Yes​ | 11 (26.2)​ | 2 (6.5)​ | 1 (33.3)​ | ​ |
| No​ | 31 (73.8)​ | 28 (90.3)​ | 2 (66.7)​ | ​ |
| Unknown​ | 0 (0.0)​ | 1 (3.2)​ | 0 (0.0​ | ​ |
| ^a^ N (%)​  ^b^ Statistical comparison was between Ipilimumab plus Nivolumab and anti-PD1 groups. ​  ^b^Included: Myasthenia-like syndrome, Encephalitis, Nephritis, Headaches, Nausea, Fatigue and Pruritus.​ | | | | |

| **Supplementary Table 5: Surgical Outcomes & Adverse Events – Nodal Surgery​** | | |
| --- | --- | --- |
| **Category**​ | **TLND^a,b^**​  **N = 32**​ | **INE^a^**​  **N = 32** |
| Lymphedema requiring PT referral​ | 9 (28.1)​ | 0 (0.0)​ |
| Seroma​ | 5 (15.6)​ | 5 (15.6)​ |
| Post-op Drain​ | 30 (93.8)​ | 6 (18.8)​ |
| Drain in place > 2 weeks​  ​ | 15 (46.9)​ | 1 (3.1)​ |
| Post-operative infection requiring IV antibiotics​ | 4 (12.5)​ | 0 (0.0)​  ​ |
| ^a^ N (%)​  ^b^ Upfront and reflex TLND were performed in 25 and 7 patients, respectively.​  ​  *Abbreviations: INE, Index Lymph Node Excision; TLND, total lymph node dissection.*​ | | |
